# Emerging perspectives on osteonecrosis of the femoral head: the role of circular RNAs and long non-coding RNAs - a systematic review

**DOI:** 10.3389/fgene.2025.1549684

**Published:** 2025-06-04

**Authors:** Feifei Lin, Shicheng Zhou, Min Yi, Qingyu Wang

**Affiliations:** Department of Orthopedics of the Second Hospital of Jilin University, Changchun, Jilin, China

**Keywords:** lncRNA, circRNA, ONFH, BMSCs, BEMCs

## Abstract

**Background:**

Osteonecrosis of the femoral head (ONFH) is a prevalent and challenging orthopedic condition that often leads to hip pain and dysfunction. Long non-coding RNAs (lncRNAs) and circular RNAs (circRNAs) have emerged as potent regulators of gene expression that influence both transcriptional and post-transcriptional processes in ONFH pathogenesis. This study aimed to investigate the association between dysregulated lncRNAs and circRNAs and their functions in ONFH.

**Methods:**

We performed a systematic literature review of PubMed, MEDLINE, and Web of Science for all publicly available data. We included papers published before 17 April 2024, to evaluate the regulatory role and differential expression of lncRNAs and circRNAs in ONFH.

**Results:**

Forty-four eligible studies were retrieved from PubMed, MEDLINE, and Web of Science, including 19 expression profiling studies, 19 gene studies, and six therapeutic studies. A total of 37 circRNAs and 42 lncRNAs were identified using quantitative real-time PCR (qRT-PCR). Dynamic changes in lncRNA and circRNA expression are associated with the proliferation and apoptosis of bone marrow stem cells (BMSCs), bone marrow endothelial cells (BMECs), and necrotic bone tissues in ONFH. CircHIPK3 and circHGF act as miRNA sponges to disrupt the osteogenic-adipogenic equilibrium, whereas lncRNA SNHG1 and GAS5 directly suppress osteogenesis. Notably, HOX transcript antisense intergenic RNA (HOTAIR), LncAABR07053481, Miat, and LINC00473 play significant roles in ameliorating the abnormal differentiation of BMSCs and could be promising therapeutic targets for ONFH.

**Conclusion:**

This systematic review discusses the current understanding of the involvement of lncRNAs and circRNAs in ONFH pathogenesis. Despite these promising findings, the limitations include heterogeneity in the study design and insufficient *in vivo* validation. This work consolidates ncRNA-mediated pathways in ONFH, offering novel targets for early diagnosis and RNA-based therapies, while advocating standardized multi-omics approaches in future research.

## 1 Introduction

ONFH results in substantial labor loss because of its high disability rate (Cui et al., 2016). Newly diagnosed cases of ONFH have remained relatively constant, with approximately 20,000 to 30,000 people affected each year in the United States, primarily among young adults aged between 20 and 40 years (Moya-Angeler et al., 2015; George and Lane, 2022). The condition typically progresses, leading to intense pain; ultimately, total hip arthroplasty becomes necessary for treating the advanced stages of ONFH (Wang et al., 2014a). ONFH is a complex pathological process, in which the interplay between various intrinsic and extrinsic factors leads to lesions in the intramedullary microvasculature. Following thrombosis, the femoral head becomes undernourished, resulting in osteoclast death. Recent findings have indicated that epigenetic regulatory mechanisms in MSCs significantly influence steroid-induced ONFH (SONFH) (Huang et al., 2023). However, the mechanisms underlying ONFH progression remain largely unclear, hindering progress in the diagnosis and therapeutic intervention of early-stage ONFH. Elucidating the underlying mechanisms represents a significant research focus that can inform strategies for the prevention, diagnosis, and treatment of ONFH.

Non-coding RNAs (ncRNAs) are vital regulatory factors in numerous biological processes such as cell death and disease pathogenesis (Liu et al., 2020). Studies have shown that ncRNAs, such as circular RNAs (circRNAs) and long non-coding RNAs (lncRNAs), are involved in the modulation of apoptosis, RNA pathways, and cell death regulation (Su et al., 2016). It is noteworthy that approximately 1.5% of the human genome is dedicated to protein-coding regions, with a vast expanse of the remaining sequences transcribed into non-coding RNAs that lack the capacity for protein coding (Lander, 2011). Although the contributions of ncRNAs to ONFH progression are increasingly being recognized owing to advances in high-throughput sequencing technologies and corresponding analytical methods (Wang A. et al., 2018; Xiang et al., 2020), a thorough evaluation of the relevant literature is yet to be conducted. Accordingly, we present an overview of the role of ncRNAs (especially circRNAs and lncRNAs) in the pathogenesis of non-traumatic ONFH.

## 2 Methods

### 2.1 Literature search protocol and search strategy

A systematic search of PubMed, MEDLINE, and Web of Science was performed for all publicly available data from the inception of the databases until 17 April 2024. The keywords and MeSH terms in our search strategy were the following keywords and combinations: “(lncRNA OR long non-coding RNA OR long ncRNA OR circular RNA OR circRNA OR ciRNA) AND (avascular necrosis OR aseptic necrosis OR osteonecrosis) AND (femoral head)”. We did not impose restrictions on the year of publication, publication status, or language, and we did not limit our inclusion to any specific study design; randomized and non-randomized clinical trials, cohort studies, and case-control studies were all considered. Additionally, we screened all articles referenced in the selected studies to identify the relevant literature. The study selection process was independently conducted by two authors (Wang and Lin), and any discrepancies were resolved through discussion.

### 2.2 Inclusion criteria and exclusion criteria

The inclusion criteria for this study were as follows: (a) investigations that obtained lncRNA and/or circRNA expression in patients with ONFH; (b) studies utilizing bone tissue, serum, plasma, or blood samples from individuals with ONFH; and (c) studies conducted on animals or ONFH cell lines. The exclusion criteria were as follows: (a) systematic reviews or meta-analyses, (b) studies without quantitative real-time PCR (qRT-PCR) to measure the expression of lncRNAs and/or circRNAs, and (c) correspondence to editors, technical notes, opinion-based studies, and single case reports. Abstracts from conferences that did not provide grouping information or sample sizes were excluded, as were those representing full-text articles already included in this study.

### 2.3 Data extraction and synthesis

Two authors (FFL and SCZ) independently evaluated the eligibility of the full-text articles and gathered essential information in a standardized format. lncRNA and circRNA data were obtained from the GEO database, microarray, and high-throughput sequencing (RNA-seq) datasets. The regulatory functions of lncRNAs and circRNAs were also considered. Other important information included the type of sample (bone tissue, serum, and plasma), the direction of lncRNA/circRNA expression (upregulated or downregulated), target miRNAs, and target mRNAs. Any inconsistencies in the data were addressed through discussions with a third review author (QYW).

## 3 Results

### 3.1 Summary of investigations into the dysregulated lncRNAs and circRNAs in ONFH

**FIGURE 1:**
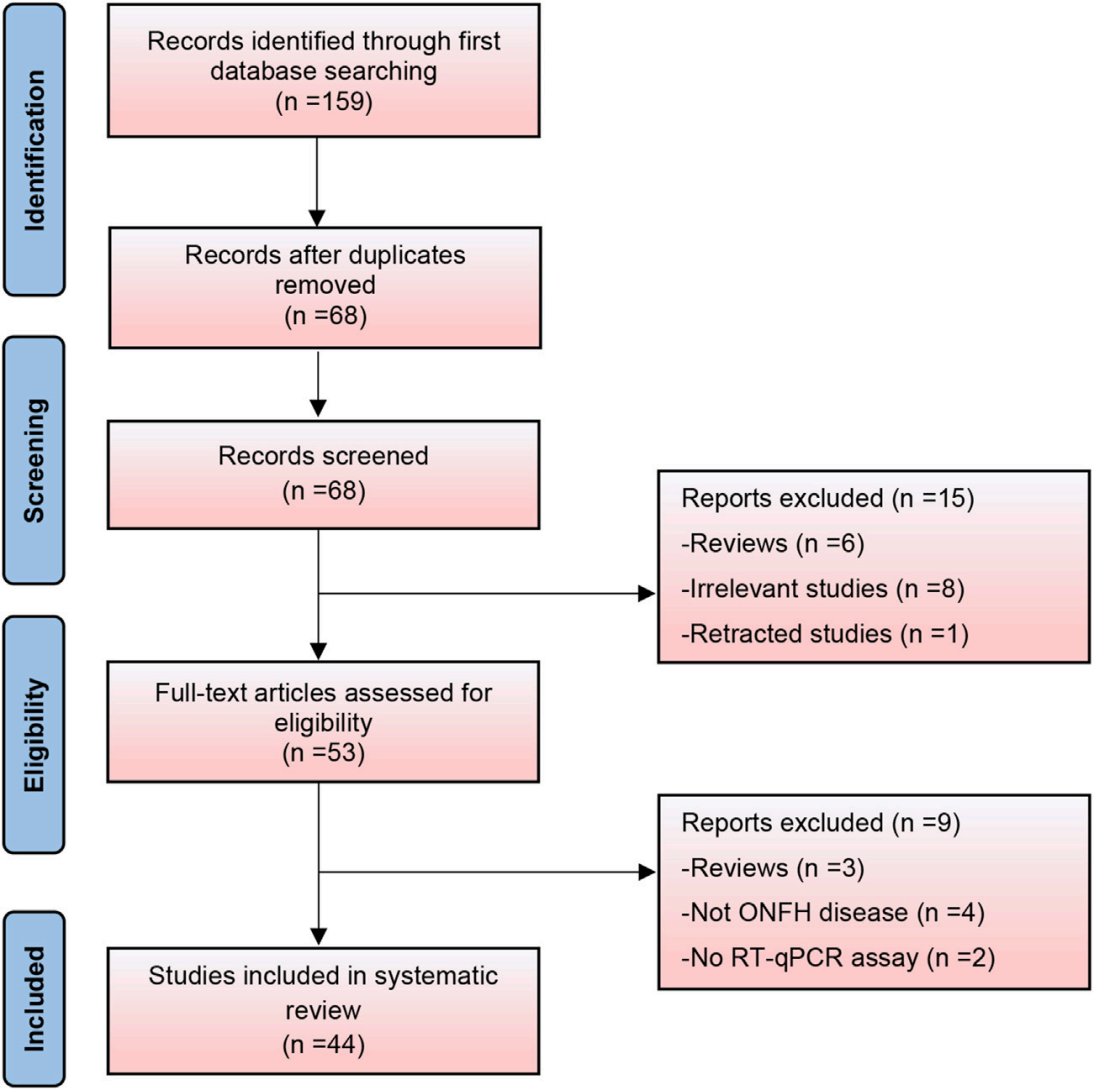
PRISMA flowchart of the study selection process.

Based on the search strategy, 159 relevant studies were initially included. After removing duplicate records, 68 studies were analyzed. Following a thorough review of titles and abstracts, 15 studies were deemed ineligible and excluded. A comprehensive full-text review was conducted on the remaining 53 records, leading to the exclusion of an additional nine studies that failed to meet the predetermined inclusion criteria. Finally, 44 studies were included in this systematic review (Figure 1). It encompassed 19 expression profiling studies, 19 specific gene studies, and 6 therapeutic studies. Apart from two studies that reported the downregulation of lncRNA metastasis-associated lung adenocarcinoma transcript 1 (MALAT1) and one study that reported its upregulation, our systematic review identified 37 circular RNAs (26 upregulated and 11 downregulated) and 41 long non-coding RNAs (20 upregulated and 21 downregulated). Thirty-one studies were related to steroid-induced ONFH, two were associated with alcohol-induced ONFH, one with trauma-induced ONFH, four with non-traumatic ONFH (no details provided), and six did not specify the type of ONFH.

### 3.2 CircRNAs expression profiles in ONFH

Seven studies, which included the validation of 27 circRNAs by qRT-PCR, revealed differentially expressed circRNAs in patients with ONFH. Of these, 18 circRNAs were upregulated and nine were downregulated (Table 1). These included four high-throughput sequencing studies and three microarray assays. Three studies focused on the differentially expressed circRNAs in bone marrow stem cells (BMSCs) of ONFH patients, two studies investigated the circRNA expression profile in the bone tissues of ONFH patients, and two studies focused on circRNA expression in peripheral blood.

**TABLE 1:** CircRNAs and lncRNAs expression profiles in ONFH.

Method	Samples (number)	Upregulation	Downregulation	qRT-PCR validated (up/down)	References
High-throughput sequencing	Subchondral bone (3v3)	44 circRNAs	30 circRNAs	circ_0001187 (up) circ_0008928 (up)	Jiao et al. (2021)
Microarray assays	BMSCs (5v5)	108 circRNAs	74 circRNAs	circHGF (up)	Feng et al. (2022)
High-throughput sequencing	BMSCs (5v5)	141 circRNAs	90 circRNAs	circ_0000219 (down) circ_0005936 (down) circ_0004588 (down)	Xiang et al. (2020)
High-throughput sequencing	Venous blood (6v6)	234 circRNAs	148 circRNAs	circRNA_25487 (up)	Zhang Y. et al. (2021)
Microarray assays	BMSCs (3v3)	460 circRNAs	360 circRNAs	circ_0003596 (up) circ_0001946 (up) circ_0020303 (up) circ_0001873 (up) circ_0000740 (up) circ_0000625 (down) circ_0084140 (down) circ_0041150 (down) circ_0050898 (down) circ_0005133 (down)	Chen G. et al. (2020)
High-throughput sequencing	Peripheral blood (3v3)	299 circRNAs	116 circRNAs	circ_0002737 (up) circ_0097391 (up) circ_0140628 (up) circ_0003344 (up) circ_0000069 (up) circ_0004692 (up) circ_0058919 (up) circ_0093677 (up)	Zhu et al. (2019)
Microarray assays	Bone tissues (3v3)	433 circRNAs	214 circRNAs	circ_0008136 (up) circ_0074758 (down)	Yao et al. (2020)
High-throughput sequencing	Subchondral bone (3v3)	126 differentially expressed lncRNAs		lncRNA GAS5 (down)	Liu et al. (2022)
High-throughput sequencing	Bone tissues	575 lncRNAs	27 lncRNAs	lncRNA FAM201A (down)	Huang et al. (2018)
Microarray assays	BMSCs (3v3)	1878 lncRNAs	1842 lncRNAs	HOTAIR (up) OGFR-AS1 (up) LOC100505817(up) RP1-67K17.3 (up) CTD-2006O16.2 (up) RP11-193H18.2 (down) XXBAC-BPGBPG55C20.3 (down) MALAT1 (down) CTD-3080F16.3 (down) RUNX1-IT1 (down)	Wang A. et al. (2018)
High-throughput sequencing	BMSCs (7v7)	181 lncRNAs	391 lncRNAs	RP11-154D6 (down)	Xiang et al. (2019)
Microarray assays	Bone tissues (3v3)	1,179 lncRNAs	3,214 lncRNAs	NR_027293 (up) T318776 (down) NR_038891 (down) ENST00000565178 (down)	Luo et al. (2019)
Microarray assays	BMSCs (3v3)	24 lncRNAs	24 lncRNAs	AC107070.1 (up) linc-ANKRD20A1-4 (up) LINC00473(down) MAPT-AS1 (down)	Li G. et al. (2021)
Microarray assays	Hip articular cartilage (4v4)	15 lncRNAs	13 lncRNAs	C9orf163 (up)	Han and Li (2021)
Microarray assays	Rat BMSCs	79 lncRNAs	72 lncRNAs	TCONS_00036420 (up) TCONS_00083120 (up) TCONS_00041960 (down) XR_085692.2 (down)	Li et al. (2016)
Microarray assays	BMSCs (3v3)	24 lncRNAs	24 lncRNAs	LINC00473(down)	Xu et al. (2020)
High-throughput sequencing	Femoral head tissues	40 abnormal expressed lncRNAs		LINC00612(up)	Li L. et al. (2021)

Subchondral bone alterations have been reported to involve a cascade of gene expression changes, signaling pathway modifications, microarchitectural shifts, and histopathological variations that culminate in structural transformations across the entire joint (Zhang et al., 2012; Li et al., 2013; Chen et al., 2017). Regarding ONFH, although related research is limited, previous studies have characterized the degeneration of the hip articular cartilage associated with ONFH (Magnussen et al., 2005; Sonoda et al., 2017; Chen et al., 2019). Histological alterations in articular cartilage, including chondrocyte depletion, surface fibrillation, subchondral bone thickening, and elevated osteoclastic activity, can lead to differential gene expression within the articular cartilage (Im et al., 2000; Wang et al., 2014b; Liu et al., 2016). This suggests that these structural alterations are not merely physical deformities but also reflect underlying molecular shifts, indicating a more complex interplay between cartilage degradation and genetic regulation. Despite extensive research, the precise pathogenesis of ONFH in the subchondral bone remains elusive. Consequently, the subchondral bone is a significant area of study that provides an opportunity to directly reflect localized pathological changes associated with the disease.

Using next-generation sequencing, 74 differentially expressed circRNAs and 121 differentially expressed mRNAs were identified in the subchondral bone of patients with ONFH compared with the intertrochanteric region of patients with femur fractures (Jiao et al., 2021). The protein-protein interaction (PPI) network analysis revealed mRNAs with high connectivity degrees, including Collagen type I alpha 1 (COL1A1), Collagen type I alpha 2 (COL1A2), bone gamma carboxyglutamate protein (BGLAP), specificity protein-7(SP7), matrix metalloprotease 9 (MMP9), secreted protein acidic and rich in cysteine (SPARC) and insulin-like growth factor 1(IGF1). The interactions examined in the subchondral bone of ONFH patients play critical roles. circ_0000551 was proposed as a competing endogenous RNA (ceRNA) of hsa-miR-526b-5p and miR-6809-5p, which targets anoctamin-5 (ANO5). circ _0008928 and circ_0003915 have been proposed as ceRNAs of miR-150-5p, miR-500b-3p, and miR-619-5p, which target chromatin-modified protein 4C (CHMP4C). circ _0008928 acts as a sponge for miR-3605-5p by targeting the integrin-binding sialoprotein (IBSP) gene. However, these ceRNA mechanisms have not been empirically validated.

One study of the expression profiles in the osteonecrotic zone versus the normal zone of ONFH revealed that 647 circRNAs were differentially regulated, with 433 circRNAs highly expressed and 214 exhibiting low expression (Han et al., 2022). circ_0058122 expression was significantly elevated in dexamethasone (Dex)-treated human umbilical vein endothelial cells (HUVECs). Dex-induced apoptosis in HUVECs was reduced when circ_0058122 was silenced, whereas it increased in cells overexpressing hsa_circ_0058122. Furthermore, hsa_circ_0058122 functioned as a ceRNA for hsa-miR-7974, which targets and interacts with insulin-like growth factor binding protein 5 (IGFBP5).

### 3.3 Regulatory role of CircRNAs in BMSCs of ONFH

Steroid-induced endothelial dysfunction disrupts the blood supply to the femoral head, leading to the progressive upregulation of osteoclast-related proteins and localized bone tissue ischemia and necrosis (Maruyama et al., 2018; Wang Q. et al., 2018; Chen G. et al., 2020). The degradation of bone cells, coupled with an imbalance between osteogenic and osteoclastic activities, ultimately leads to deterioration and collapse of the bone structure (Piuzzi et al., 2019). Disruption of the BMSC differentiation equilibrium indicates a significant pathological alteration in this process (Powell et al., 2011; Chang et al., 2020). BMSCs with heightened adipogenic potential not only lose their regenerative capabilities but also result in the catastrophic accumulation of adipocytes and elevated internal bone pressure in the femoral head, thereby worsening the advancement of SONFH.

**FIGURE 2:**
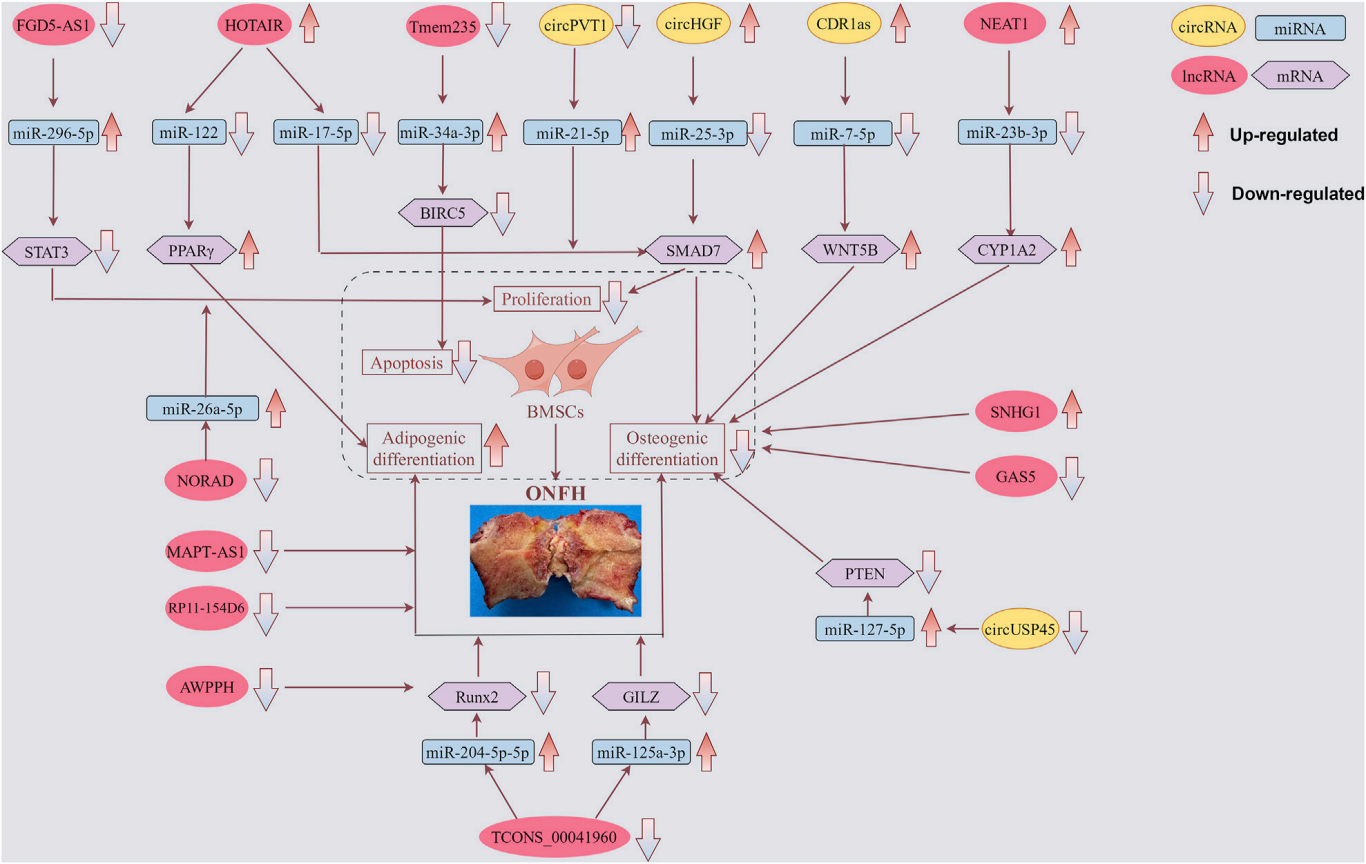
Functions of specific circRNAs and lncRNAs in proliferation, apoptosis, adipogenic and osteogenic differentiation of BMSCs. LncRNAs FGD5-AS1, HOTAIR, Tmem235, NEAT1, NORAD, and TCONS_00041960, as well as circRNAs CDR1as, circPVT1, circHGF, and circUSP45, regulate BMSCs through corresponding miRNAs, thereby contributing to the progression of ONFH. Meanwhile, lncRNAs MAPT-AS1 and RP11-154D6 directly promote adipogenic differentiation, whereas lncRNAs SNHG1 and GAS5 directly inhibit osteogenic differentiation. The yellow box symbolizes circRNA. The red box symbolizes lncRNA. The blue box symbolizes miRNA. The purple box symbolizes mRNA.

As shown in Table 2 and Figure 2, four circRNAs (circPVT1, circUSP45, circHGF, and CDR1as) regulated BMSCs. These circRNAs primarily affect cell proliferation and osteogenic differentiation. One study identified 182 differentially expressed circRNAs in ONFH-BMSCs, of which 108 were upregulated and 74 were downregulated (Feng et al., 2022). Additionally, among the upregulated circRNAs, circHGF was shown to suppress both the proliferation and osteogenic differentiation of BMSCs in ONFH by targeting the miR-25-3p/SMAD7 axis. Chen K. et al. (2020) screened circRNAs using microarray assays and identified 820 differentially expressed circRNAs in SONFH BMSCs. These included 460 upregulated and 360 downregulated circRNAs. Additionally, they found that the circRNA CDR1as was overexpressed in SONFH-BMSCs, which led to decreased osteogenic differentiation through the CDR1as-miR-7-5p-WNT5B axis.

**TABLE 2:** Functions of the circRNAs in ONFH.

Name	Expression	p Value	Functional role	Target miRNAs	Target genes	Species	Sample	References
circHGF	Up	<0.01	Suppress proliferation and osteogenic differentiation	miR-25–3p	SMAD7	Human	BMSCs	Feng et al. (2022)
CDR1as	Up	<0.001	decrease osteogenic and increase adipogenic differentiation	miR-7-5p	WNT5B	Human	BMSCs	Chen K. et al. (2020)
circRNA_25487	Up	<0.05	inhibits bone repair	miR-134–3p	p21	Human	Venous blood	Zhang C. et al. (2021)
circHIPK3	Down	<0.001	promote BMECs proliferation, migration and angiogenesis	miR-7	KLF4/VEGF	Human	BMECs	Peng et al. (2023)
circ_0058122	Up	<0.001	Promote HUVECs apoptosis	miR-7974	IGFBP5	Human	HUVECs	Yao et al. (2022)
CDR1as	Up	<0.001	suppress BMECs activity and angiogenesis	miR-135b	FIH-1	Human	BMECs	Mao et al. (2021)
circPVT1	Down	<0.05	promote osteogenesis and apoptosis	miR-21–5p	SMAD7	Rats	BMSCs	Hao et al. (2021)
circUSP45	Up	<0.05	inhibit osteogenesis	miR-127–5p	PTEN	Human	BMSCs	Zhou Y. et al. (2021)
circ_0058792	Up	data not shown	decrease ALP activity and osteogenic differentiation	miR-181a-5p	SMAD7	Rats	MC3T3-E1 and HEK-293 T	Han et al. (2022)
CDR1as	Up	<0.001	positively associated with ARCO classification	——	——	Human	plasma and local necrotic tissues	Jiang et al. (2020)

In one study, the circRNA expression profile of ONFH-BMSCs was determined using high-throughput sequencing. This analysis revealed 231 differentially expressed circRNAs, of which 141 were upregulated and 90 were downregulated. Of these, 215 circRNAs were derived from exonic regions, with only 16 originating from intronic or intergenic regions (Xiang et al., 2020). Intriguingly, the present study confirmed the time-dependent expression patterns of circ_0000219, circ_0004588, circ_0005936, miR-144-3p, and miR-1270 during osteogenic and adipogenic differentiation of BMSCs.

In addition to investigating the expression profiles of ONFH-BMSCs, studies have also been conducted on the regulatory roles and functions of specific circRNAs. The upregulation of circ_0066523 observed during the osteogenic induction of BMSCs indicated its role in promoting proliferation and differentiation by epigenetically silencing phosphatase and tensin homolog (PTEN), thereby activating the AKT pathway (Xin et al., 2021). This discovery paves the way for the identification of therapeutic targets relevant to osteoblast differentiation disorders such as ONFH.

### 3.4 Regulatory role of CircRNAs in BMECs of ONFH

Recent studies indicate that glucocorticoid-induced dysfunction of BMECs may result in changes in the microcirculation of the femoral head, potentially contributing significantly to the development of SONFH (Kerachian et al., 2009). BMECs modulate apoptosis during angiogenesis. An inverse relationship has been reported among angiogenic function, vascular integrity, and the extent of apoptosis. Prolonged exposure to glucocorticoids has been linked to the inhibition of angiogenesis, induction of cell apoptosis, and impairment of endothelial cell function (O'Connell and Genest Jr, 2001; Williams et al., 2006; Zhang et al., 2016). Previous research has indicated a decline in the angiogenic potential of BMECs coupled with an elevated propensity for apoptosis in patients with SONFH (Yu et al., 2020). Considering that ONFH involves compromised blood supply to the femoral head, circRNAs that govern angiogenesis could be critical. Altered expression of these circRNAs may impede the development of new blood vessels, subsequently affecting the delivery of essential nutrients and oxygen to the bone tissue.

Circular RNA homeodomain-interacting protein kinase 3 (circHIPK3) is a prevalent circular RNA that functions as a miRNA sponge, thereby modulating cellular processes, including angiogenesis, proliferation, migration, and apoptosis (Fu and Sun, 2021). CircHIPK3 is also involved in several pathophysiological processes, including fibrosis, tumorigenesis, and vascular endothelial damage (Zhou J. et al., 2021). In patients with ONFH, circHIPK3 expression was downregulated in necrotic tissue (M–W U test, U = 0, p < 0.001). Upregulation of circHIPK3 augmented the proliferative, migratory, and angiogenic capabilities of BMECs, while reducing their rate of apoptosis. However, these effects were reversed upon introduction of the miR-7 mimic (Peng et al., 2023).

In addition to its function in BMSCs, the influence of circCDR1as on angiogenesis in ONFH-associated BMECs has also been explored. The migration of BMECs was significantly enhanced in the circCDR1as silencing group compared to that in the negative control group. Furthermore, transfection with circCDR1as plasmids upregulated the protein expression of hypoxia inducible factor 1 (FIH-1) (p < 0.05), and conversely, decreased the expression of hypoxia inducible factor-1α (HIF-1α) and vascular endothelial growth factor (VEGF) compared to the NC group (p < 0.05) (Mao et al., 2021).

### 3.5 Regulatory role of plasma circRNAs in ONFH

The role of plasma circRNAs in ONFH is an emerging area of research that aims to elucidate the molecular mechanisms underlying this debilitating condition. In previous studies, significant alterations in specific mRNAs such as MMP9, Alpha-2-Macroglobulin, and CXC motif chemokine ligand 12 (CXCL12)/stromal cell-derived factor 1 (SDF-1) were detected in the serum of patients with ONFH (Ghale-Noie et al., 2018; Zheng et al., 2022; Li et al., 2023). These modifications in gene expression are instrumental in evaluating the severity of ONFH. However, studies focusing on circRNAs in the serum of ONFH patients are scarce.

Studies of plasma and local circRNA expression have garnered significant interest. This study enrolled ninety-nine patients diagnosed with nontraumatic ONFH and ninety-nine healthy individuals. Elevated expression of circCDR1as was observed in both the plasma and local necrotic tissues using RT-qPCR (Jiang et al., 2020). Additionally, this study suggests a positive correlation between the expression of both plasma and local circCDR1as and the Association Research Circulation Osseous (ARCO) classification system. Receiver operating characteristic (ROC) curve analysis implied that plasma levels of circCDR1as could serve as a potential biomarker for monitoring radiographic progression in patients with non-traumatic ONFH.

### 3.6 Research on lncRNA expression profiles in ONFH

Ten studies, which included the validation of 31 lncRNAs by qRT-PCR, revealed differentially expressed lncRNAs in patients with ONFH. Of these, 15 lncRNAs were upregulated, and 16 were downregulated (Table 1). These included four high-throughput sequencing studies and six microarray assays. Five studies focused on the differentially expressed lncRNAs in the BMSCs of patients with ONFH, and five studies investigated the lncRNA expression profile in the bone or cartilage tissues of patients with ONFH.

Using high-throughput RNA sequencing, Liu et al. (2022) detected the expression levels of lncRNAs and mRNAs in subchondral bone samples obtained from three patients diagnosed with ONFH and three with femoral neck fractures (FNF). A total of 126 and 959 differentially expressed lncRNAs and genes, respectively, were identified. More importantly, the expression of lncRNA GAS5 was strongly correlated with the osteogenic differentiation of BMSCs. This association was further substantiated by the notable downregulation of GAS5 in the subchondral trabecular bone tissue of patients diagnosed with ONFH and in ONFH rat models. The observed downregulation was statistically significant, as indicated by a log2FoldChange value of less than −1 and a p-value of 0.003. This study posits that lncRNA GAS5 could serve as an osteogenic biomarker for ONFH, offering a potential target for the early diagnosis and molecular therapy of ONFH. Huang et al. (2018) identified 575 upregulated and 27 downregulated lncRNAs in ONFH samples using RNA sequencing. The lncRNA FAM201A was significantly downregulated in ONFH samples compared with that in FNF samples, and the expression levels of FAM201A were found to be correlated with the progression of ONFH. Xiang et al. (Xiang et al., 2019) identified 181 upregulated and 391 downregulated lncRNAs in BMSCs derived from patients with steroid-induced ONFH using RNA sequencing analysis. Notably, the expression of lncRNA RP11-154D6 was significantly reduced in BMSCs from patients with ONFH. This reduction was associated with the promotion of osteogenic differentiation and inhibition of adipogenic differentiation in BMSCs.

In one of our previous studies utilizing microarray analysis (Wang A. et al., 2018), we reported that 1878 lncRNAs were significantly upregulated, whereas 1842 lncRNAs demonstrated statistically significant downregulation in BMSCs from patients with SONFH compared to the control group. The coding-non-coding co-expression (CNC) network and ceRNA network analyses indicated that lncRNA RP11-193H18.2, metastasis-associated lung adenocarcinoma transcript 1 (MALAT1), and HOX transcript antisense intergenic RNA (HOTAIR) were associated with abnormal osteogenic and adipogenic differentiation of BMSCs in patients with steroid-induced SONFH. However, these regulatory relationships were not experimentally validated in this study. Additionally, using microarray analysis, Luo et al. (2019) revealed that there were 1,179 upregulated and 3,214 downregulated lncRNAs in the bone tissues of steroid-induced ONFH. The upregulated lncRNAs (NR_027293) and downregulated lncRNAs (T318776, NR_038891, and ENST00000565178) in the ONFH group were validated by RT-qPCR. Li T. et al. (2020) identified 24 downregulated and 24 upregulated lncRNAs in the BMSCs of patients with ONFH using microarray analysis. Among these dysregulated lncRNAs, the overexpression of MAPT antisense RNA 1 (MAPT-AS1) was observed to enhance osteogenesis while suppressing adipogenesis in BMSCs, as evidenced at both the cellular and mRNA levels.

### 3.7 Regulatory role of lncRNAs in BMSCs of ONFH

The role of lncRNAs in ONFH BMSCs is an intricate area of research that is yet to be fully understood. The significance of lncRNAs in the epigenetic regulation of BMSCs is becoming increasingly apparent as they potentially influence both osteogenic and adipogenic differentiation (Suh et al., 2005; Sheng et al., 2007; Xu et al., 2022). Dysregulation of these long non-coding transcripts may contribute to the development and progression of ONFH by altering normal cellular pathways that maintain bone health and remodeling. Further investigations into the specific mechanisms by which lncRNAs affect the differentiation of BMSCs may offer significant insights into the pathogenesis of ONFH. This may facilitate the development of innovative therapeutic strategies targeting these molecular pathways.

As shown in Table 3 and Figure 2, 11 lncRNAs (3 upregulated and 8 downregulated) have been reported to regulate BMSCs. These lncRNAs primarily affect cell proliferation, apoptosis, osteogenic differentiation, and lipogenic differentiation. HOTAIR, the first identified trans-acting lncRNA, has been observed to exhibit abnormally high expression levels in various tumor tissues and cell lines. These cancers include gastric cancer, breast cancer, hepatic carcinoma, ovarian cancer, and acute myeloid leukemia (Wu et al., 2014). Using microarray analysis, Xing et al. identified HOTAIR expression in osteoarthritic cartilage. Furthermore, its expression was significantly higher than that in normal cartilage samples (Xing et al., 2014). Wei et al. confirmed that HOTAIR suppressed miR-17-5p expression to regulate osteogenic differentiation and proliferation by interacting with miR-17-5p and SMAD7 (Wei et al., 2017). Le et al. (2023) demonstrated that the HOTAIR/miR-122/PPARγ signaling pathway mediated alcohol-induced ONFH in a rat model. Additionally, they found that sustained downregulation of miR-122 expression was responsible for the ongoing progression of alcohol-induced ONFH, even after cessation of alcohol intake.

**TABLE 3:** Functions of the lncRNAs in ONFH.

Name	Expression	p Value	Functional role	Target miRNAs	Target genes	Species	Sample	References
MALAT1	Up	<0.05	increase osteoclast differentiation	miR-329–5p	PRIP	Rat	Femoral head tissues	Li G. et al. (2021)
GAS5	Down	0.003	promote osteogenic differentiation	——	——	Human	BMSCs	Liu et al. (2022)
MALAT1	Down	<0.05	promote osteogenic differentiation	miR-214	ATF4	Human	Femoral head tissues	Huang et al. (2020)
HOTAIR	Up	<0.01	inhibit osteogenic differentiation and proliferation	miR-17–5p	SMAD7	Human	BMSCs	Wei et al. (2017)
SNHG1	Up	<0.001	inhibit osteogenic differentiation	——	——	Human	BMSCs	Zhang C. et al. (2021)
Tmem235	Down	<0.05	inhibit hypoxia-induced apoptosis	miR-34a-3p	BIRC5	Rat	BMSCs	Zhang et al. (2022)
HOTAIR	Up	<0.01	promote adipogenic differentiation	miR122	PPARγ	Rat	BMSCs	Le et al. (2023)
Miat	Up	<0.05	promote osteogenesis	——	——	Human	osteonecrotic tissue	Fang et al. (2019)
FAR591	Up	<0.001	decrease angiogenesis and proliferation	——	——	Rat	BMEC	Zhang et al. (2023)
TCONS_00041960	Down	<0.01	promote osteogenesis and inhibit adipogenesis	miR-204–5p miR-125a-3p	Runx2 GILZ	Rat	BMSCs	Shang et al. (2018)
RP11-154D6	Down	<0.01	promote BMSCs osteogenesis and inhibit adipogenesis	——	——	Human	BMSCs	Xiang et al. (2019)
DGCR5	Up	<0.05	repress BMSCs nuclear localization of b-catenin	——	Rac1 inactivated peptide	Human	BMSCs	Jiang et al. (2023)
NORAD	Down	<0.01	promotes differentiation and proliferation	miR-26a-5p	——	Human	BMSCs	Fu et al. (2021)
MAPT-AS1	Down	<0.05	promote osteogenic differentiation and inhibit adipogenic differentiation	——	——	Human	BMSCs	Li T. et al. (2020)
NEAT1	Up	<0.05	inhibit osteogenic differentiation	miR-23b-3p	CYP1A2	Human	BMSCs	Zhou J. et al. (2021)
AWPPH	Down	<0.05	inhibit expression of Runx2	——	Runx2	Human	BMSCs	Chen X. et al. (2020)

In the context of ONFH, BMSCs are affected by various factors that can lead to reduced proliferation and increased apoptosis, contributing to the development and progression of this debilitating condition (Qi and Zeng, 2015; Wu F. et al., 2019). For instance, decreased proliferation of BMSCs can limit the ability of the bone to repair itself, leading to a weakened bone structure and potential collapse of the femoral head. Concurrently, an increase in BMSC apoptosis diminishes the pool of viable cells essential for bone regeneration. Dex suppresses the proliferation of human BMSCs and promotes apoptosis in a dose-dependent manner. Overexpression of FGD5-AS1 promoted cell proliferation and suppressed apoptosis in hBMSCs treated with Dex. Furthermore, FGD5-AS1 functions as a molecular sponge of miR-296-5p. Additionally, miR-296-5p directly targets the signal transducer and activator of transcription 3 (STAT3). Importantly, both miR-296-5p and STAT3 modulated the effects of lncRNA FGD5-AS1 on hBMSCs treated with Dex (Wu et al., 2022). These findings highlight the complex interplay between lncRNAs, miRNAs, and signaling pathways in regulating the response of hBMSCs to glucocorticoid treatment, which could have implications for therapeutic strategies targeting bone health and repair.

### 3.8 Regulatory role of lncRNAs in BMECs of ONFH

LncRNAs expressed in BMECs play a pivotal role in angiogenesis, which is crucial for maintaining the integrity of the femoral head. Angiogenesis, the process of forming new blood vessels from pre-existing ones, plays a crucial role in supplying essential nutrients and oxygen to the bone tissue (Li Z. et al., 2020). Dysregulated lncRNA expression in BMECs can impair angiogenesis, potentially leading to bone death, which is a defining characteristic of ONFH. Furthermore, glucocorticoids (GCs) have been found to induce apoptosis in BMECs and impede vascular regeneration (Zuo et al., 2020; Huang et al., 2021). Such occurrences precipitate dysfunction in bone microcirculation, thereby disrupting the synchrony between bone microcirculation and osteogenesis. This chain of events culminates in osteogenic damage and the onset of osteonecrosis.

Characterization of the expression profiles of lncRNAs in ONFH-BMECs is important. Zhang et al. (2023) conducted lncRNA/mRNA microarray and bioinformatics analyses using a model of GC-induced apoptosis in BMECs. A total of 105 lncRNAs exhibiting concentration-dependent expression changes owing to GC were identified, of which 46 were upregulated and 59 were downregulated. Among these, FAR591 was markedly upregulated during GC-induced BMEC apoptosis and femoral head necrosis (p < 0.001). Targeted deletion of FAR591 effectively abrogated GC-induced apoptosis of BMECs, thereby mitigating the deleterious effects of GCs on the femoral head microcirculation and inhibiting the pathogenesis and progression of steroid-induced ONFH.

### 3.9 Regulatory role of plasma lncRNAs in ONFH

Plasma lncRNAs have been identified as potential biomarkers for ONFH diagnosis and prognosis. The expression profiles of lncRNAs in the plasma of patients with ONFH differed significantly from those of healthy controls, suggesting their involvement in disease pathogenesis. Further research is necessary to elucidate the role of plasma lncRNAs in ONFH and their clinical utility as ONFH biomarkers.

Initially, MALAT1 was identified as a significant predictor of lung cancer (Ji et al., 2003). Previous studies have revealed that MALAT1 is significantly downregulated during compromised osteogenic differentiation of BMSCs in patients with steroid-induced ONFH. Furthermore, MALAT1 is capable of safeguarding human osteoblasts from dexamethasone-induced damage by engaging the Ppm1e-AMPK signaling pathway (Fan et al., 2018; Wang Q. et al., 2018). In SONFH, MALAT1 enhances osteogenic differentiation by modulating the activation of transcription factor 4 (ATF4) through the sequestration of miR-214 (Huang et al., 2020). Jin et al. (2021) further confirmed the significant downregulation of MALAT1 in both plasma and femoral head necroses. Notably, 104 patients with non-traumatic ONFH and 100 healthy controls were included in this study. The authors suggested that reduced levels of serum and local MALAT1 expression could potentially reflect disease severity in patients with nontraumatic ONFH.

AWPPH is a recently identified lncRNA that plays an oncogenic role in the development of hepatocellular and bladder cancers (Zhao et al., 2017; Zhu et al., 2018). Chen X. et al. (2020) were the first to report that AWPPH expression was significantly downregulated in patients with non-traumatic ONFH compared to healthy controls, as observed in both BMSCs and serum samples. Importantly, the authors proposed that AWPPH might contribute to ONFH development by enhancing the expression of Runx2.

### 3.10 Regulatory role of lncRNAs in ONFH therapy

In addition to the aforementioned investigations, six studies explored the treatment of ONFH by modulating lncRNAs. Two studies have reported that neohesperidin ameliorates ONFH by regulating the lncRNAs SNHG1 and HOTAIR (Yuan et al., 2020; Zhang C. et al., 2021). The other four studies investigated the regulation of lncRNAs (LncAABR07053481, Miat, and LINC00473) on abnormal differentiation of BMSC, which alleviates avascular necrosis (Fang et al., 2019; Xu et al., 2021; 2022; Wang et al., 2023). LINC00473 was found to be significantly downregulated in BMSCs and exhibited a protective role against dexamethasone-induced apoptosis by modulating the PEBP1/Akt/Bad/Bcl-2 signaling pathway (Xu et al., 2020; 2021). Transplantation of polylactic-co-glycolic acid (PLGA) hydrogels loaded with rat-derived BMSCs modified by LINC00473 significantly enhanced bone repair and regeneration in the necrotic region of the femoral head in a SONFH rat model (Xu et al., 2022). This discovery highlights the potential of LINC00473 as a molecular mediator with therapeutic relevance in mitigating glucocorticoid-induced osteonecrosis.

Huo Xue Tong Luo capsule (HXTL), a traditional Chinese medicinal formulation, has been found to significantly mitigate pain and halt the progression of necrosis, leading to short-to mid-term joint collapse. This effect results in a decreased rate of joint collapse, reduced need for total hip arthroplasty (THA), and fewer symptoms compared with the natural history of ONFH (Wei et al., 2019). Furthermore, a chromatin immunoprecipitation (ChIP) assay revealed that the HXTL capsule significantly enhanced the enrichment of H3K27me3 and diminished the presence of H3K4me3 in the promoter regions of lncRNA-miat (Fang et al., 2019). This indicates that the HXTL capsule suppressed lncRNA-miat expression through histone modification. Taken together, the HXTL capsule may foster osteogenesis to improve ONFH, at least in part, by suppressing the transcriptional expression of Miat.

## 4 Discussion

The refinement of microarray assays and high-throughput sequencing detection technologies, coupled with advanced data analysis techniques, ensures high readability of data (Shoemaker et al., 2001; Salzman et al., 2012; Zhao et al., 2020). This enables the swift retrieval of information from an extensive array of samples, facilitating their extensive use across diverse sectors, including genetic studies, pharmacodynamics, and disease diagnosis and treatment. In recent years, research involving high-throughput sequencing and gene chips pertinent to ONFH has increased, significantly propelling investigations into the etiology of the disease and unveiling the critical regulatory functions of lncRNAs. This systematic review elucidates the emerging roles of circRNAs and lncRNAs in ONFH, a significant orthopedic condition with a complex etiology. ONFH is characterized by bone cell death due to ischemia, which leads to structural failure and articular surface collapse if left untreated. Despite extensive research, the molecular mechanisms underlying ONFH remain unclear, hindering the development of targeted therapies.

Our findings highlight that circRNAs and lncRNAs are not merely transcriptional byproducts but also play critical regulatory roles in the cellular processes implicated in ONFH. CircRNAs arise from the splicing of one or two exons. This splicing event is characterized by the covalent bonding of the 3′and 5′ends, leading to the formation of a covalently closed, continuous loop. This unique structure sets circRNAs apart from other RNA species and contributes to their stability in cells (You et al., 2015; Chen et al., 2016; Greene et al., 2017). The functional roles of circRNAs are likely intertwined with their distinctive stabilities. Notably, previous studies have suggested that circRNAs can function as miRNA sponges, sequester miRNAs, or interact with functional proteins to regulate specific biological processes at either the transcriptional or post-transcriptional level (Hansen et al., 2013; Memczak et al., 2013; Müller and Appel, 2017). In this review, circPVT1, circUSP45, circHGF, and CDR1as were shown to regulate BMSC differentiation by acting as sponges.

LncRNAs can act as epigenetic regulators by binding to DNA or modifying histones, altering the chromatin structure, and affecting gene expression patterns (Xia et al., 2014; Shields et al., 2019). This may lead to the dysregulation of genes involved in bone metabolism and vascular health, contributing to bone tissue necrosis. HOTAIR, FGD5-AS1, Tmem235, NEAT1, NORAD, and TCONS_00041960 function as ceRNAs and influence the expression of genes related to osteogenesis and adipogenesis, key processes implicated in ONFH. LncRNAs can modulate signaling pathways that are essential for maintaining bone homeostasis, such as the Wnt/β-catenin, Hedgehog, and Notch pathways (Wu X. et al., 2019; Ma et al., 2021; Zhang X. et al., 2021; Zhao et al., 2023). The dysfunction of these pathways due to aberrant lncRNA activity can disrupt the equilibrium between bone formation and resorption, leading to the onset of ONFH.

The implications of these findings are twofold. First, they expand our understanding of ONFH beyond the traditional risk factors and provide a foundation for exploring novel therapeutic targets. Targeted interventions aimed at modulating specific circRNAs or lncRNAs could offer more precise and effective treatment options than the current empirical approaches. Second, given the unique expression patterns of these RNA species, they hold promise as diagnostic tools for the early detection and monitoring of ONFH, potentially enabling personalized medicine strategies. The dysregulated circRNAs and lncRNAs identified here hold promise as noninvasive biomarkers for early ONFH detection. Furthermore, RNA-targeted therapies such as antisense oligonucleotides or nanoparticle-delivered mimics can restore the balance of BMSC differentiation, offering alternatives to invasive surgical interventions.

## 5 Conclusion

Currently, advancements in the life sciences are deeply interconnected with the development of biodetection technologies. Over the past decade, extensive research has revealed significant roles of lncRNAs and circRNAs in numerous diseases. LncRNAs and circRNAs perform intricate regulatory functions in ONFH, suggesting their potential as novel diagnostic and therapeutic targets. Future research is needed to systematically identify these RNA molecules and their interaction networks, elucidate their specific mechanisms in the development of ONFH, and evaluate their effectiveness as biomarkers or therapeutic tools. However, it is also evident that there is a paucity of studies on the therapeutic effects of lncRNAs and circRNAs in ONFH, which should indeed constitute a significant focus for future research. Current studies are constrained by the small sample size, heterogeneity in ONFH subtypes, and the lack of *in vivo* validation of the proposed ceRNA networks. Future studies should prioritize the use of multicenter cohorts and functional assays. The conclusion now acknowledges translational barriers, such as RNA stability in clinical samples and off-target effects of RNA-based therapies. Further research is expected to provide more personalized and precise medical solutions for patients with ONFH.
